# Comparative study of different surgical approaches for treatment of UPJ obstruction according to the degree/severity of hydronephrosis factor

**DOI:** 10.3389/fped.2022.966292

**Published:** 2022-08-04

**Authors:** Peng Zhao, Cao Wang, Kaiyi Mao, Zhen Luo, Yingbo Li, Guangxu Zhou, Hongyang Tan, Hong Liu, Yucheng Mao, Hong Ma, Xianhui Shang, Bin Liu

**Affiliations:** ^1^Guizhou Children's Hospital, Zunyi, China; ^2^Department of Pediatric Surgery, Affiliated Hospital of Zunyi Medical University, Zunyi, China

**Keywords:** retroperitoneal laparoscope, transperitoneal laparoscope, UPJO, children, pyeloplasty

## Abstract

**Objective:**

To compare the efficacy of two different surgical approaches during and after pyeloplasty according to the degree/severity of hydronephrosis factor.

**Materials and methods:**

Sixty child patients with UPJ obstruction admitted to our hospital from August 2019 to October 2021 were collected. Patients who underwent retroperitoneal laparoscopic pyeloplasty (RPLP) were enrolled into Group A (*n* = 20), while those who received transperitoneal laparoscopic pyeloplasty (TLP) were selected as Group B (*n* = 40). Clinical parameters, including gender, age, laterality of UPJ obstruction, degree/severity of hydronephrosis, body weight, operation time, drainage tube indwelling time, complete oral feeding time, and length of hospital stay, were compared between the two groups.

**Results:**

All 60 child patients were operated upon successfully without conversion to open surgery. There were no statistically significant differences in gender, age, laterality of UPJ obstruction, and body weight between the two groups, while the operation time of TLP was shorter than that of RPLP, indicating a statistically significant difference (*P* < 0.001). The differences in complete oral feeding time, drainage tube indwelling time, and length of hospital stay were statistically significant between the two groups, and RPLP was superior to TLP in terms of postoperative recovery time (*P* < 0.001). A stratified comparison showed that there were no statistically significant differences in anteroposterior diameter ≤ 20 mm, while there were statistically significant differences in anteroposterior diameter >20 mm. Hydronephrosis is reviewed after 3 months of the operation, degree/severity of hydronephrosis have been reduced.

**Conclusion:**

Both RPLP and TLP are safe and feasible in the treatment of UPJ obstruction, and their overall surgical effects are equivalent. For child patients with anteroposterior diameter ≤ 20 mm, RPLP is available, while patients with anteroposterior diameter >20 mm, TLP is recommended.

## Introduction

Pediatric hydronephrosis mainly resulting from ureteropelvic junction (UPJ) obstruction can be treated by the Anderson-Hynes operation, which is viewed as the gold standard (1). Currently, laparoscopic surgery has become the preferred way to treat UPJ obstruction, which is principally categorized into retroperitoneal laparoscopic pyeloplasty (RPLP) and transperitoneal laparoscopic pyeloplasty (TLP) (2–6). This study's aim is to analyze and summarize the efficacy of these two surgical approaches during and after pyeloplasty to validate their security and feasibility for the treatment of UPJ obstruction.

## Materials and methods

### Patients and data

Clinical data of 60 child patients with UPJ obstruction admitted to our hospital from August 2019 to October 2021 were collected for retrospective analysis. The diagnostic standard used in this study are: ultra sound (SFU classification) (7, 8), intravenous urography (IVU), magnetic resistance urography (MRU), Emission Computed Tomography (ECT) confirm repeatedly obstructed curves on the diuresis renogram and impaired split renal function <40%. All patients were unilateral UPJO. Exclusion criteria were bilateral UPJO requiring intervention, vesicoureteral reflflux, obstructive primary megaureter, ureterocele, posterior urethral valve or the existence of other structural anomalies. This study retrospectively analyzed 60 cases: the RPLP group A (*n* = 20) and the TLP group B (*n* = 40). Clinical parameters including gender, age, laterality of UPJ obstruction, body weight, degree/severity of hydronephrosis, operation time, drainage tube indwelling time, complete oral feeding time, and length of hospital stay were compared between the two groups.

### Surgical methods

Preoperative routine preparation, gastrointestinal preparation, indwelling of urethral catheter, and general anesthesia with endotracheal intubation were given to patients. An experienced Pediatric surgeon have done all the operations used in this study.

**Figure 1:**
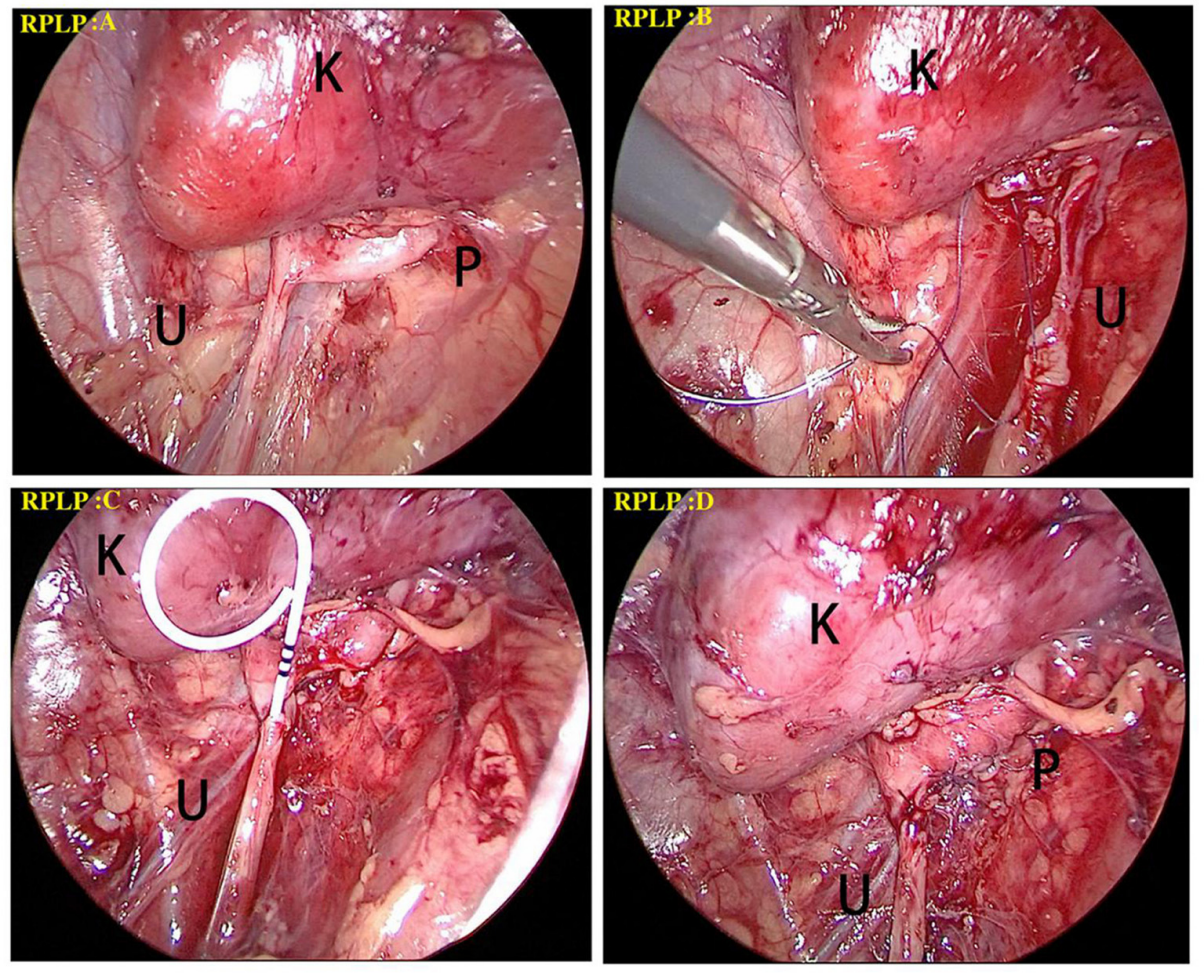
Surgical procedure of RPLP for the treatment of a left-side UPJO. **(A)** The perirenal fascia was longitudinally cut to expose the dorsal side of the lower pole of the kidney (black K), followed by separation and exposure of the renal pelvis (black P) and upper ureter (black U) to clarify the location and cause of stenosis. **(B)** The anastomosis started between the vertex of spatulated ureter (black U) and the most dependent part of renal pelvis. **(C)** A double J tube was antegradely introduced through the anastomosis. **(D)** The remainder of anastomosis was completed.

RPLP was performed in Group A. The patient was placed in a lateral position on the unaffected side with the waist padded on the operating table. First, a 1.5-cm skin incision was made at 1–2 transverse fingers above the iliac spine at the axillary midline. Next, hemostatic forceps were used to bluntly separate muscles, lumbodorsal fascia, and the psoas major muscle, with the index finger moving closely along to the location below the 12th rib at the posterior axillary line, followed by placing a 0.5-cm trocar. The index finger pushed the anterior peritoneum back to the ventral side and then pressed against and below the costal arch at the axillary front line, followed by placing a 0.5-cm trocar, and then a 0.5-cm trocar was inserted above the iliac spine with silk thread fixing the muscle and skin. Subsequently, the perirenal fascia was longitudinally cut to expose the dorsal side of the lower pole of the kidney, followed by separation and exposure of the renal pelvis and upper ureter to clarify the location and cause of stenosis. Then the stenosis portion was resected completely, and a double-J tube was placed after anastomosis (2, 3, 9). At the end of the operation, a retroperitoneal drainage tube was indwelled, followed by closing the incision (Figure 1).

**Figure 2:**
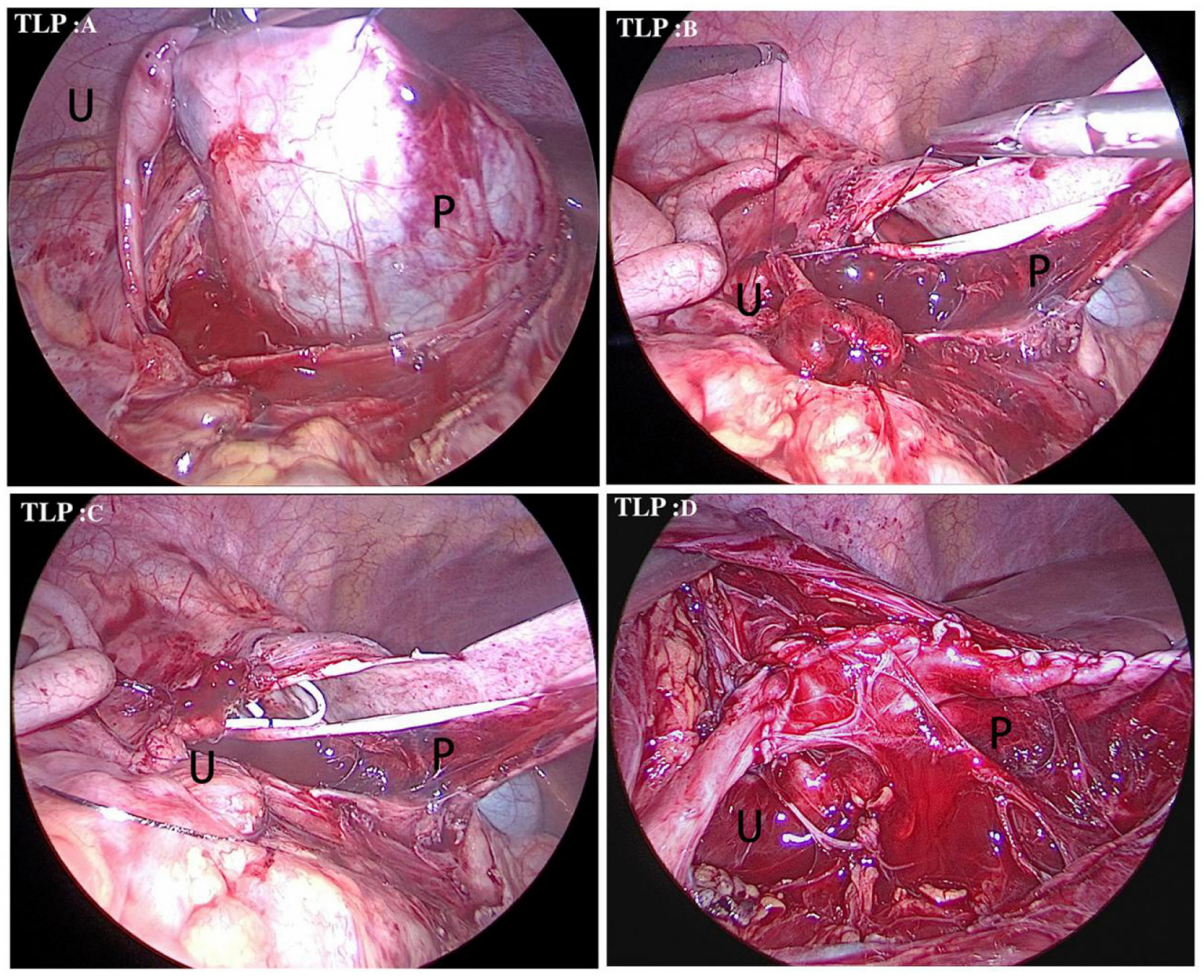
Surgical procedure of TLP for the treatment of a right-side UPJO. **(A)** Identifification of the distended renal pelvis (black P) through a paracolonic sulcus. The upper pole of the renal pelvis was suspended and upper ureter (black U) to clarify the location. **(B)** The anastomosis started between the vertex of spatulated ureter (black U) and the most dependent part of renal pelvis (black P). **(C)** A double J tube was antegradely introduced through the anastomosis. **(D)** The remainder of anastomosis was completed.

TLP was performed in Group B. The patient was placed on the operating table with the waist padded on the affected side. Three 0.5-cm trocars were inserted into the umbilicus (midline of the abdomen), 2.0 cm away from the upper and lower umbilicus margins, and were arranged in a straight line. Surgical treatment was carried out through the paracolonic sulcus, which is located at the lower edge of the mesenteric vein. The medial edge of the colon was left free to fully expose the paracolonic sulcus, and the perirenal fascia was open at the lateral edge of the genital vein to expose the lower pole of the kidney and to expand the renal pelvis, followed by separation and exposure of the renal pelvis and upper ureter to clarify the location and cause of stenosis. The upper pole of the renal pelvis was suspended and dilated. Then the stenosis portion was resected, and a double-J tube was indwelled after anastomosis. At the end of operation, an abdominal drainage tube was placed into the pelvic cavity (4, 5, 10), and then the incision was closed (Figure 2).

### Statistical analysis

SPSS 25.0 software was adopted for statistical analysis. Categorical variables were compared by a chi-square test. Continuous variables were subjected to a normality test. Those normally distributed data were expressed by the mean ± standard deviation and compared by an independent-samples *t*-test, while those not conforming to normal distribution were expressed as the median and interquartile ranges (lower quartile—upper quartile) and compared by an independent-samples non-parametric test.

## Results

All 60 patients were operated upon successfully without conversion to open surgery. The results of comparative analysis revealed that mean average age, operation time, drainage tube indwelling time, complete oral feeding time, and length of hospital stay were [51 (36.5–96) months vs. 41.5 (23.75–63.75) months], [152.5 (146.25–182.5) min vs. 140 (126.25–150) min], [29 (27.25–30) h vs. 40 (38–41.75) h], [(40.65 ± 2.89) h vs. (50.05 ± 3.94) h], and [6 (5–6) d vs. 7 (6–7) d], respectively, between Groups A and B. No patients required blood transfusion during and after the operation in the two groups. Among the patients in Group A, one suffered abdominal pain and fever following removal of a double-J tube after operation. When anastomotic stenosis was proven by inspection, a secondary operation was performed and the patient recovered well postoperatively.

**Figure 3:**
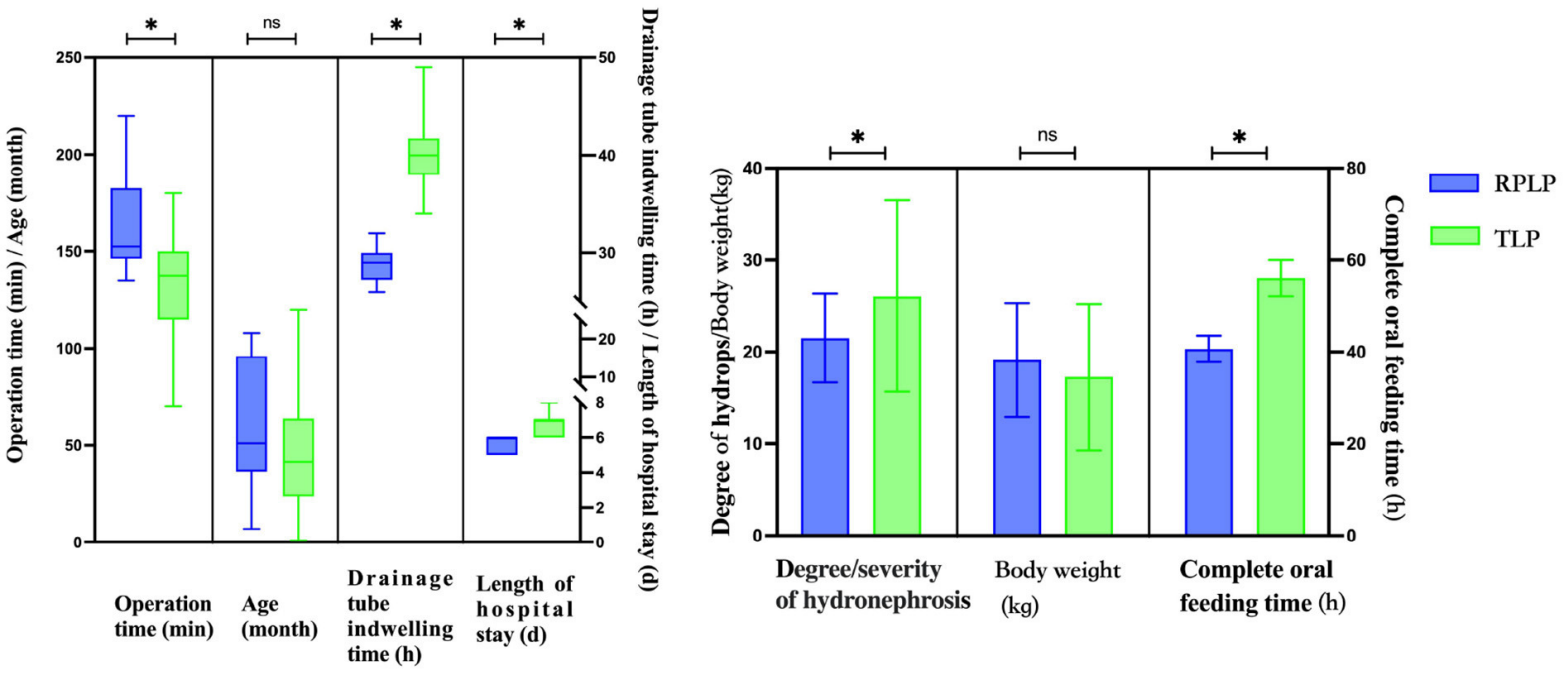
Comparisons of clinical specific differences between RPLP and TLP. “ns” means no significant difference, *p <0.05.

There were no statistically significant differences in gender, age, laterality of UPJ obstruction, and body weight between the two groups, while the operation time of TLP was shorter than that of RPLP, indicating a statistically significant difference (*P* = 0.001). The differences in complete oral feeding time, drainage tube indwelling time, and length of hospital stay were statistically significant between the two groups, and RPLP was superior to TLP in terms of postoperative recovery time (*P* < 0.001; Table 1; Figure 3). Stratified comparison of the degree/severity of hydronephrosis showed that in the case of hydronephrosis with an anteroposterior diameter >20 mm, the operation time of TLP was shorter than that of RPLP, indicating a statistically significant difference (*P* < 0.001), while the differences in complete oral feeding time, drainage tube indwelling time, and length of hospital stay were statistically significant between the two groups, and RPLP was superior to TLP in terms of postoperative recovery time (*P* < 0.001). In the case of hydronephrosis with an anteroposterior diameter ≤ 20 mm, there was no statistically significant difference in operation time between the two surgical approaches (*P* = 0.701), while the differences in complete oral feeding time, drainage tube indwelling time, and length of hospital stay were statistically significant between the two groups, and RPLP was superior to TLP in terms of postoperative recovery time (*P* < 0.01; Table 2). In comparison to the pre-operation situation, hydronephrosis is reviewed after 3 months of the operation, Ultrasound examination showed that degree/severity of hydronephrosis have been reduced to determine the success of the operations.

**Table 1:** Comparisons of clinical outcomes between RPLP and TLP.

		**Surgical approach**		**χ^2^/Z**	***P***
		**RPLP**	**TLP**		
Gender^Δ^	Male	12 (27.90%)	31 (72.10%)	2.011	0.156
	Female	8 (47.10%)	9 (52.90%)		
Laterality of UPJ obstruction^Δ^	Left	15 (34.90%)	28 (65.10%)	0.164	0.685
	Right	5 (29.40%)	12 (70.60%)		
Degree/severity of hydronephrosis^#^		21.5 ± 4.85	26.05 ± 10.42	−2.306	0.025
Age (month)^Δ^		51 (36.5–96)	41.5 (23.75–63.75)	−1.46	0.144
Body weight (kg)^#^		19.1 ± 6.22	17.24 ± 7.98	0.913	0.365
Operation time (min)^Δ^		152.5 (146.25–182.5)	140 (126.25–150)	−3.22	0.001
Complete oral feeding time (h)^#^		40.65 ± 2.89	56.05 ± 3.94	−15.489	<0.001
Drainage tube indwelling time (h)^Δ^		29 (27.25–30)	40 (38–41.75)	−6.298	<0.001
Length of hospital stay (d)^Δ^		6 (5–6)	7 (6–7)	−4.899	<0.001

Δ *SW normality test indicates data not conforming to normal distribution*.

# *SW normality test indicates data conforming to normal distribution*.

**Table 2:** Comparisons of clinical outcomes between RPLP and TLP according to different degree/severity of hydronephrosis.

		**Surgical approach**		**χ^2^/Z**	***P***
		**RPLP**	**TLP**		
>20 mm	Operation time (min)^#^	163.18 ± 24.32	132.93 ± 18.30	4.259	<0.001
	Complete oral feeding time (h)^#^	40.09 ± 2.66	56.24 ± 3.91	−12.596	<0.001
	Drainage tube indwelling time (h)^Δ^	28 (27~30)	40 (37–42)	−4.854	<0.001
	Length of hospital stay (d)^Δ^	6 (5–6)	6 (6–7)	−3.83	<0.001
≤ 20 mm	Operation time (min)^#^	161.67 ± 24.75	158.18 ± 10.31	0.395	0.701
	Complete oral feeding time (h)^#^	41.3 ± 3.16	55.55 ± 4.18	−8.405	<0.001
	Drainage tube indwelling time (h)^#^	29.00 ± 1.87	39.10 ± 1.70	−12.626	<0.001
	Length of hospital stay (d)^Δ^	6 (5.5–6)	7 (6–7)	−2.982	0.003

Δ *SW normality test indicates data not conforming to normal distribution*.

# *SW normality test indicates data conforming to normal distribution*.

## Discussion

UPJ obstruction is one of the common diseases in the Department of Pediatric Urology, and it can be effectively treated by surgery (11). This study is the first to feature a comparison of RPLP and TLP according to the degree/severity of hydronephrosis before operation, and it can serve as a supplement to the selection of preoperative surgical regimens. First, a stratified comparison was conducted for the degree/severity of hydronephrosis. Considering that most renal functions have been damaged to varying extents in the case of severe hydronephrosis with an anteroposterior diameter > 20 mm, and the postoperative recovery is poorer in such children than those with an anteroposterior diameter ≤ 20 mm, the degree/severity of hydronephrosis of 20 mm was selected as a cutoff value for research.

Pediatric hydronephrosis is mainly caused by ureteropelvic junction stenosis. In addition, such factors as high insertion of the ureter, ureteral calculi, ureteral polyps, ureteral valves, reduced peristaltic function of the ureteropelvic junction and upper ureter, abnormal fibrous cord and nerve vascular compression outside the ureter, and retrocaval ureter can also induce hydronephrosis. The Anderson-Hynes operation, which is regarded as the gold standard for the treatment of UPJ obstruction (1), can be categorized into four types according to different surgical approaches: open dismembered pyeloplasty, laparoscopic dismembered pyeloplasty, robot-assisted laparoscopic pyeloplasty, and endoscopic incision of or balloon dilatation in ureteral stenosis. As surgical operations have continuously developed in refinement, mechanization, and visualization, open dismembered pyeloplasty has usually use infants and after repeated failures of laparoscopic surgery (12–14). Although robot-assisted laparoscopic pyeloplasty can achieve good results, it can not be widely carried out due to economic reasons and difference in medical levels (15–17). As for endoscopic incision or balloon dilatation for the treatment of ureteral stenosis, considering that hydronephrosis easily recurs because of ureteral scarring caused by thermal injury owing to the holmium laser, as well as the uncertain effect of balloon dilatation, accompanied by the risk of bleeding, urinary extravasation and ureteral rupture, such surgical methods with a low success rate are rarely used in the clinic (18, 19). At present, most regions UPJ obstruction is primarily treated by laparoscopic dismembered pyeloplasty, which is principally categorized into RPLP and TLP according to the surgical approach (20, 21).

Most pediatric urologists prefer the transperitoneal approach. It is more invasive, yet there are advantages such as a larger operation space, better exposure of the surgical field, a more familiar dissection approach, and a more intuitive anastomotic operation. But there are also some disadvantages: (A) the gastrointestinal gut will be influenced for long time operation; (B) the complete healing time is longer than that of the retroperitoneal laparoscopic pyeloplasty; (C) there is a certain possibility of internal hernia after the operation (22). The results of this study demonstrate that the transperitoneal approach exhibits notable advantages in the treatment of UPJ obstruction in patients with severe hydrops (with an anteroposterior diameter > 20 mm). However, little attention has been paid by pediatric urologists to the retroperitoneal laparoscopic approach, especially for infant patients, which may be attributed to the narrow operation space, insufficient exposure of the surgical field, unfamiliar dissection approach, and difficult surgical position. In fact, the success rate, bleeding volume, and complications display no significant differences between the two surgical approaches for the treatment of urinary diseases in adult patients (23–26). This study reveals that the retroperitoneal laparoscopic approach is a favorable selection for child patients with mild hydronephrosis (with an anteroposterior diameter ≤ 20 mm), therefore not only avoiding excessive surgical injury but also obtaining faster recovery. However, there are also some difficulties (27). First, the peritoneum as a semi-permeable membrane is more transparent in infants, and a longer operation time may lead to a situation in which CO_2_ enters the abdominal cavity through the peritoneum, making the operation space narrower. Second, the positioning of the trocar needs to be more accurate because of the limited operation space, increasing the difficulty of the operation for beginners. Third, because of the particularity of body position, children's bodies are small and cannot be placed in the folding knife lateral position like adults to obtain greater operation space, so the waist should be padded on the unaffected side. Fourth, severe hydronephrosis can cause a narrow operation space, and skilled technology is required for anastomotic treatment, especially for dorsal pyeloureterostomy, which is not recommended for beginners. Then the stenosis portion was resected, followed by anastomosis, and the patient recovered well postoperatively. Hence, RPLP also has its own advantages (28–30): (A) It has a relatively independent operation space with less interference in the abdominal viscera. (B) The dissociation of the kidney is completed in the vessel-free area (Gerota's fascia), with relatively less bleeding. (C) The renal vessels, renal pelvis, and ureter are exposed more clearly, reducing the bleeding risk. (D) For children with mild hydronephrosis, the renal pelvis can be found more quickly. (E) Ureteral distortion can also be safely and completely free, so the tissue injury is less than that caused by the transperitoneal approach. (F) The drainage tube indwelling time and mean complete oral feeding time are shorter than those of the laparoscopic approach, indicating a faster postoperative recovery.

This was a single-center study with a small sample size. Considering the changes in the learning curve of retroperitoneal laparoscopy and the fact that retroperitoneal laparoscopy has been adopted in few hospitals in China for the treatment of UPJ obstruction, many samples are still needed for further research. Besides, given that the follow-up period in this study was short, a long-term follow-up is also needed to further investigate the postoperative outcomes. We will further improve our study by conducting multi-center experiments and increasing samples numbers to make the conclusion more accurately.

## Conclusion

Both RPLP and TLP are safe and feasible for the treatment of UPJ obstruction, and their overall surgical effects are equivalent. For child patients with mild hydronephrosis (with an anteroposterior diameter ≤ 20 mm), no notable difference is shown in the operation time of the two surgical approaches, but RPLP is superior to TLP in terms of postoperative overall recovery, so RPLP is available to treat UPJ obstruction. For child patients with severe hydronephrosis (anteroposterior diameter > 20 mm), the operation time of TLP is significantly shorter than that of RPLP, so TLP is recommended to treat UPJ obstruction.

## Data availability statement

The raw data supporting the conclusions of this article will be made available by the authors, without undue reservation.

## Ethics statement

Written informed consent was obtained from the individual(s), and minor(s)' legal guardian/next of kin, for the publication of any potentially identifiable images or data included in this article.

## Author contributions

Study conception and design and critical revision: BL. Data acquisition: CW, KM, ZL, GZ, and YL. Analysis and data interpretation: HT, HL, YM, HM, and XS. Drafting of the manuscript: PZ. All authors contributed to manuscript revision, read, and approved the submitted version.

## Funding

This study was supported by the National Natural Science Foundation of China (Grant No. 82060277), the Guizhou Provincial Health Committee Support Program (Grant No. gzwjkj2019-1-005), and the Doctoral Startup Fund of the Affiliated Hospital of Zunyi Medical University, Project Number: 2020-04.

## Conflict of interest

The authors declare that the research was conducted in the absence of any commercial or financial relationships that could be construed as a potential conflict of interest.

## Publisher's note

All claims expressed in this article are solely those of the authors and do not necessarily represent those of their affiliated organizations, or those of the publisher, the editors and the reviewers. Any product that may be evaluated in this article, or claim that may be made by its manufacturer, is not guaranteed or endorsed by the publisher.
